# Effect of Human Umbilical Cord Mesenchymal Stem Cells Transplantation on Nerve Fibers of A Rat Model of Endometriosis

**DOI:** 10.22074/ijfs.2015.4211

**Published:** 2015-04-21

**Authors:** Yan Chen, Dong Li, Zhe Zhang, Natsuko Takushige, Bei-Hua Kong, Guo-Yun Wang

**Affiliations:** 1 Department of Obstetrics and Gynecology of the Third Affiliated Hospital of Zhengzhou University, Zhengzhou, Henan 450052, China; 2 Cryomedicine Laboratory, Qilu Hospital of Shandong University, Jinan 250012, China; 3 Department of Obstetrics and Gynecology, Qilu Hospital of Shandong University, Jinan 250012, China; 4 Department of Obstetrics and Gynaecology, University of Sydney, Sydney, NSW, 2006, Australia

**Keywords:** Endometriosis, Mesenchymal Stem Cells, Nerve Fibers, Immunohistochemistry

## Abstract

**Background:**

Endometriosis is a common, benign, oestrogen-dependent, chronic gynaecological disorder associated with pelvic pain and infertility. Some researchers have identiﬁed nerve ﬁbers in endometriotic lesions in women with endometriosis. Mesenchymal stem cells (MSCs) have attracted interest for their possible use for both cell and gene therapies because of their capacity for self-renewal and multipotentiality of differentiation. We investigated how human umbilical cord-MSCs (hUC-MSCs) could affect nerve ﬁbers density in endometriosis.

**Materials and Methods:**

In this experimental study, hUC-MSCs were isolated from fresh human umbilical cord, characterized by flow cytometry, and then transplanted into surgically induced endometriosis in a rat model. Ectopic endometrial implants were collected four weeks later. The specimens were sectioned and stained immunohistochemically with antibodies against neuroﬁlament (NF), nerve growth factor (NGF), NGF receptor p75 (NGFRp75), tyrosine kinase receptor-A (Trk-A), calcitonin gene-related peptide (CGRP) and substance P (SP) to compare the presence of different types of nerve ﬁbers between the treatment group with the transplantation of hUC-MSCs and the control group without the transplantation of hUC-MSCs.

**Results:**

There were significantly less nerve fibers stained with specific markers we used in the treatment group than in the control group (p<0.05).

**Conclusion:**

MSC from human umbilical cord reduced nerve ﬁber density in the treatment group with the transplantation of hUC-MSCs.

## Introduction

Endometriosis is defined as the presence of tissues which somewhat resembles endometrial glands and stroma outside the uterine cavity, most commonly implanted over visceral and peritoneal surfaces within the female pelvis. Endometriosis exhibits disturbances of cellular proliferation, cellular invasion and neoangiogenesis (1). Although the exact prevalence of endometriosis in the general population is not clear, the prevalence in women of reproductive age is estimated to range between 10 and 15% (2). Endometriosis is a chronic, benign, oestrogen- dependent multifactorial and gynaecological disease, with pain being the most common and specific symptom. To date, the cardinal treatments for endometriosis are medical and surgical therapies. Pain symptoms may persist despite seeming adequate medical or/and surgical treatment of the disease (3).

Stem cell therapy as a promising and unprecedented strategy has the potential to be more effective than single-agent drug therapies (4). Mesenchymal stem cells (MSCs) are especially well suited for cell therapy owing to their ability to differentiate into different lineages and secrete a number of cytokines (5). Human umbilical cord-MSCs (hUC-MSCs) have become strong candidates for a cell-based therapy because of their key characteristics of long-term self-renewal and capacity to differentiate into diverse tissues. In addition, they can be easily obtained and cultured without raising ethical issues (6), as well as being an excellent alternative to bone marrow as a source of MSCs for cell therapies (6, 7). Furthermore, hUC-MSCs are a subset of primitive stem cells. HUC-MSCs neither induce teratomas nor result in acute rejection after being transplanted into non-immune- suppressed animals (8). In various animal disease models, transplantation of hUC-MSCs was reported to improve neurobehavioral functions following ischemic stroke (9), ameliorate mouse hepatic injury (10), and show effectiveness in apomorphine-induced rotations in a rodent model of Parkinson’s disease (6, 11). Nevertheless, currently little is known about the application of hUC-MSCs to endometriosis.

Some researchers have identified nerve fibers in endometriotic lesions in women with endometriosis (12-14). Berkley et al. (15) and Oosterlynck et al. (16) have reported that endometriotic implants developed a sensory and sympathetic nerve supply both in rats and in women, similar to that of the healthy rat uterus. The present study demonstrated the existence of a much greater density of nerve fibers in deep infiltrating endometriosis than in peritoneal endometriotic lesions (17). These nerve fibers in endometriotic lesions could possibly exert their functions on the pathogenesis or symptoms of endometriosis.

As a consequence, we established surgically induced endometriosis in a rat model to investigate the effects of the hUC-MSCs transplantation on nerve fibers and the pathogenesis of the disease.

## Materials and Methods

### Generation and administration of hUC-MSC

The study protocol was approved by the Research Ethics Committee of Qilu Hospital of Shandong University (Shandong, P. R. China). HUCs (n=10, clinically normal pregnancies) were excised and washed in a 0.1 mol/L phosphate buffer saline (PBS, pH=7.4, Gibco-BRL, Grand Island, NY, USA) to remove excess blood (6). The cords were dissected and the blood vessels were removed. The remaining tissues were cut into small pieces (1-2 mm^3^) and placed in plates with low-glucose Dulbecco-modified Eagle medium (L-DMEM, Gibco-BRL, Grand Island, NY, USA), supplemented with 10% fetal bovine serum (FBS, Gibco-BRL, Grand Island, NY, USA), 2 ng/mL vascular endothelial growth factor (VEGF, R&D Systems, Minneapolis, MN), 2 ng/mL epidermal growth factor (EGF, R&D Systems, Minneapolis, USA), 2 ng/ mL fibroblast growth factor (FGF, R&D Systems, Minneapolis, USA), 100 U/mL penicillin, and 100 μg/mL streptomycin (Gibco-BRL, Grand Island, USA). Cultures were maintained at 37˚C in a humidified atmosphere with 5% CO_2_. The media were changed every 3-4 days. Adherent cells proliferated from individual explanted tissues 7-12 days after initiating incubation. At this time, the small tissue pieces were removed from the culture and the adherent fibroblast- like cells were cultured to confluence, which subsequently took 2-3 weeks in culture. The cells were then trypsinized using 0.25% trypsin (Gibco-BRL, Grand Island, USA) and passaged at 1×10^4^ cells/cm^2^ in the medium described above. The cells were used after five or more passages.

### Cell surface antigen phenotyping

Fifth- to seventh-passage cells were collected and treated with 0.25% trypsin. The cells were stained with either fluorescein isothiocyanate- conjugated or phycoerythrin-conjugated monoclonal antibodies in 100 μL PBS for 15 minutes at room temperature, as suggested by the manufacturer. The antibodies used were against human antigens cluster of differentiation 34 (CD34), CD29, CD44, CD45, CD105, and CD106 (SeroTec, Raleigh, NC, USA). Cells were analyzed using flow cytometry (Cytometer 1.0, CytomicsTM FC500, Beckman Coulter Inc., USA). Positive cells were counted and compared to the signal of corresponding immunoglobulin isotypes.

### Differentiation capacity

To investigate the differentiation potential of the fibroblast-like cells, the fourth passage cells were cultured under conditions appropriate for inducing the differentiation of each lineage.

Cells were seeded at a density of 2×10^4^ cells/ cm^2^ and the differentiation media were changed every 3-4 days. The osteogenic differentiation medium consisted of L-DMEM supplemented with 10% FBS, 0.1 μM dexamethasone, 50 mM β-glycerol phosphate, and 0.2 mM ascorbic acid (Sigma-Aldrich, St. Louis, MO, USA). The adipogenic differentiation medium consisted of high-glucose DMEM supplemented with 0.25 mM 3-isobutyl-1-methylxanthine, 0.1 μM dexamethasone, 0.1 mM indomethacin (Sigma- Aldrich, USA), 6.25 μg/mL insulin (PeproTech, UK), and 10% FBS. Cells were kept in the normal growth medium served as the control.

### Animal model and cell transplantation

All animal procedures were conducted in accordance with the institutional guidelines of Qilu Hospital of Shandong University (Shandong, P. R. China). Adult female Wistar rats, weighing 180-210 g, were housed in cages in an air-conditioned room at 25 ±1˚C with a 12 hours dark/light cycle. The oestrous stage was monitored daily by vaginal smear every morning, beginning at least 2 weeks before surgery and continued until the day of death. Only rats with a regular 4-day cycle both before and after surgery were used. Surgically induced endometriosis in a rat model was done as previously described (18) and surgery was done under aseptic precautions. Rats in estrus were anesthetized with 3% pelltobarbitalum natricum (Solarbio, Beijing Solarbio Science & Technology Co., Ltd. China) at a dose of 0.2 mL /200 g by means of intraperitoneal injection. A midline abdominal incision exposed the uterus, and a 1-cm segment of the middle of the left uterine horn was removed and placed in warm sterile saline. Four pieces of uterine horn (≈2×2 mm) were cut from this segment and sewn with 4.0 nylon sutures around the alternate cascade mesenteric arteries that supply the caudal small intestine, starting from the caecum. The incision was closed in layers, and the rats were allowed to recover from anesthesia under close observation. Hereafter the endometriosis model rats were randomly divided into two groups (12 rats each), namely the treatment group and the control group. Two weeks later, the treatment group received hUC-MSCs by injection of 1×10^6^ cell/ mL normal saline into the tail vein every 5 days for 15 days. Meanwhile, the control group only received the same volume of normal saline. Four weeks later, ectopic implants were collected and fixed in 10% neutral buffered formalin for 18~24 hours.

### Immunohistochemistry

We examined the presence of different types of nerve fibers in endometriotic implants in a rat model by immunohistochemistry using highly specific markers. We used neurofilament (NF), nerve growth factor (NGF), NGF receptor p75 (NGFRp75), tyrosine kinase receptor-A (Trk- A), calcitonin gene-related peptide (CGRP) and substance P (SP) to differentiate types of nerve fibers.

These implants were fixed with formalin, processed and embedded in paraffin according to a standard protocol. Each section was cut at 4 um and mounted onto slides. These sections were routinely stained with haematoxylin and eosin (H&E, Gibco-BRL, Grand Island, NY, USA) staining. For immunohistochemistry, the slides were submitted to antigen retrieval by boiling in citrate buffer (0.01 mol/L, pH=6.0) for 15 minutes using a micro-wave oven.

Endogenous peroxidase activity was prevented by incubating in 0.3% hydrogen peroxide for 15 minutes. Nonspecific binding was blocked by 10% goat serum (Zhongshan Golden Bridge Biotecnology Co., Ltd., China) for 20 minutes at room temperature. The sections were immunostained overnight at 4˚C using antibodies for monoclonal mouse anti-NF (dilution 1:150; Abcam, UK), a highly specific marker for myelinated nerve fibers, as follows: polyclonal rabbit anti-NGF (dilution 1:200; Abcam, UK), monoclonal mouse anti-NGFRp75 (dilution 1:200; Abcam, UK), polyclonal rabbit anti-TrkA (dilution 1:500; Abcam, UK), polyclonal mouse anti-SP (dilution 1:250), and polyclonal rabbit anti-CGRP (dilution 1:300, Abcam, UK), which are sensory fiber markers, and they can be present in both Ad and C nerve fibers. The slides were washed and incubated with horseradish peroxidase-conjugated secondary antibody at room temperature for 30 minutes.

Peroxidase activity was visualized by exposure to diaminobenzidine tetrahydrochloride solution (DAB kit, Zhongshan Golden Bridge Biotecnology Co., Ltd., China) for 3-5 minutes. The sections were then washed, counterstained with hematoxylin for 1 minute, dehydrated, and mounted with coverslips. We used normal rat skin as a positive control as it reliably contains myelinated and unmyelinated nerve fibers expressing NF, NGF, NGFRp75, Trk-A, SP, and CGRP.

### Quantification of nerve fiber density

The images were captured using an Olympus DP72 camera (Tokyo, Japan). The assessment of the mean density of nerve fibers was performed by Image Pro Plus software (Media Cybernetics, MD, USA). The integrated optical density (IOD) and area of the images were calculated using Image Pro Plus software. The area was divided by integrated optical density to obtain the mean density of nerve fibers. All lighting conditions and magnifications were held constant. Moreover, the investigator was unaware of the experimental groups from which the slices were obtained.

### Statistical analysis

The results were expressed as the mean±SD. All analyses were performed using the SPSS (SPSS Inc., Chicago, IL, USA) version 17.0. The comparison between two groups was performed using non-parametric 2-tailed t test (Mann-Whitney test). Statistical significance was defined as a p value of less than 0.5.

## Results

**Fig.1:**
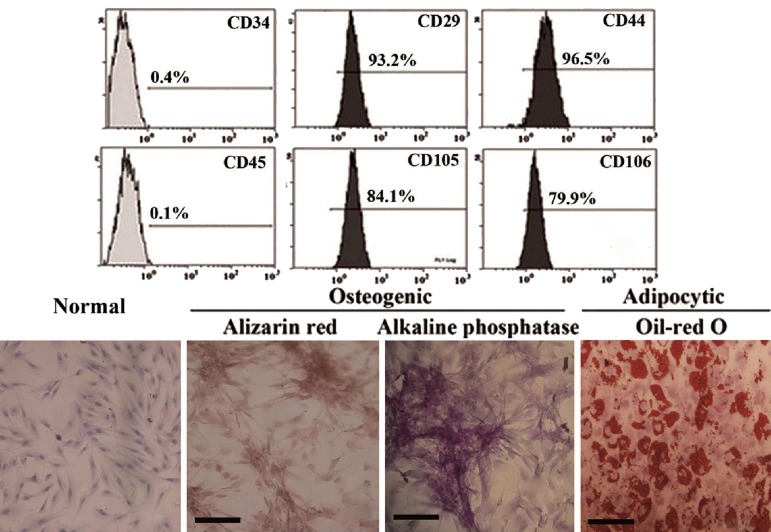
HUC-derived MSC-like cells in passaged cultures. Immunophenotype (A) and H&E staining of UC-derived MSC-like cells (B). Osteogenic differentiation as indicated by the formation of mineralized matrix shown by alizarin red staining (C) and alkaline phosphatase expression (D). Adipocytic differentiation was noted by the presence of broadened morphology and formation of lipid vacuoles (E) (positive oil-red O staining). Scale bars=80 μm. hUC; Human umbilical cord, MSCs; Mesenchymal stem cells, H&E; Haematoxylin and eosin and CD; Cluster of differentiation.

After several passages, adherent cells from UC could form a monolayer of typical fibroblastic cells. Flow cytometry results showed that UC-derived cells shared most of their immunophenotype with MSCs, including positive expression for stromal markers (CD29, CD44, CD105, and CD106), but negative expression for hematopoietic markers (CD34 and CD45) (Fig.1A, B).

MSC differentiation was assessed using the fourth passage cells. When being induced to differentiate under osteogenic conditions, MSC congregation increased with increasing induction time and formed a mineralized matrix, as confirmed by alizarin red staining (Fig.1C). Most of the MSC-like cells became alkalinephosphatase- positive by the end of 14 days (Fig.1D). No mineralized matrix was observed in the control cells kept in the normal growth medium. The spindle shape of the MSCs flattened and broadened after 1 week of adipogenic induction. Small oil droplets gradually appeared in the cytoplasm. By the end of the second week, almost all cells contained numerous oil-red-O-positive lipid droplets (Fig.1E). The control cells maintained in the regular growth medium did not stain positive for oil red O.

**Fig.2:**
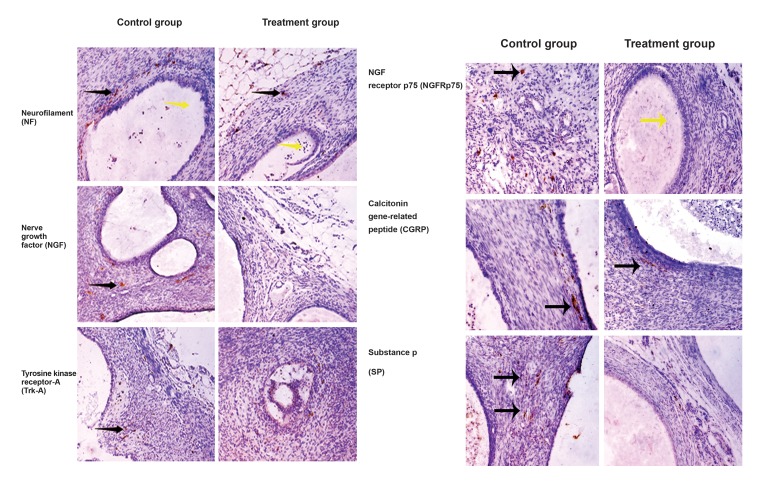
Nerve fibers in ectopic endometriotic lesions. A. Nerve fibers stained with NF from the control group without the transplantation of hUC-MSCs. B. Nerve fibers stained with NF from the treatment group with the transplantation of hUC-MSCs. C. Nerve fibers stained with NGF from the control group. D. Nerve fibers stained with NGF from the treatment group. E. Nerve fibers stained with Trk-A from the control group. F. Nerve fibers stained with Trk-A from the treatment group. G. Nerve fibers stained with NGFRp75 from the control group. H. Nerve fibers stained with NGFRp75 from the treatment group. I. Nerve fibers stained with CGRP from the control group. J. Nerve fibers stained with CGRP from the treatment group. K. Nerve fibers stained with SP from the control group. L. Nerve fibers stained with SP from the treatment group. Scale bars represent 50 μm in A-R (magniﬁcation ×200). Black arrows represent nerve fibers and yellow arrows represent endometrial glands.

The mean density values of nerve fibers are given in table 1. Nerve fibers stained with kinds of special markers in ectopic endometriotic lesions were shown in figure 2. In summary, there were significant differences (p<0.05) in the mean density of nerve fibers in endometriotic implants stained with most of the specific markers which we used between the treatment group and the control group.

**Table 1:** Quantitative assessment of the endometrial mean nerve fiber density stained against different neural markers in model rat with endometriosis


Marker	Treatment group (n=12) Mean±SD (range)	Control group (n=12) Mean±SD (range)

**NF**	0.40± 0.20 (0.19~0.90)	1.50± 1.27* (0.33~4.28)
**NGF**	0.27± 0.23 (0.00~0.77)	1.23± 0.72** (0.39~2.59)
**Trk-A**	0.19± 0.11 (0.00~0.44)	1.64± 0.95** (0.26~3.74)
**NGFRp75**	0.24± 0.13 (0.00~0.55)	0.99± 1.04* (0.17~4.07)
**CGRP**	0.32± 0.35(0.08~1.08)	1.45± 1.58* (0.33~5.64)
**SP**	0.24± 0.11 (0.00~0.45)	1.32± 1.23** (0.15~5.18)


Data are represented by mean density±SD. NF; Neuroﬁlament, NGF; Nerve growth factor, Trk-A; Tyrosine kinase receptor-A, NGFRp75; NGF receptor p75, CGRP; Calcitonin gene-related peptide, SP; Substance P, *; P<0.01 and **; P<0.001.

## Discussion

NF as a highly specific marker for myelinated nerve fibers stains Aα, Aβ, Aγ, Aδ and B fibers. Both SP and CGRP are sensory nerve fiber markers that can be present in both Aδ and C nerve fibers. In the present study, statistically significant difference was observed in the mean density of the NFimmunoactive nerve fibers between the treatment and control groups. Lower number of nerve fibers stained with NGF, TrkA, and NGFRp75 existed in the treatment group than in the control group. The mean densities of the CGRP- and SP- immunoreactive nerve fibers were lower in the treatment group, which indicates that the sensory nerve fibers were reduced. To sum up, our results showed that there were less nerve fibers stained with most of the specific markers used in this study in the treatment group compared with the control group.

It is believed that rich innervation in endometriosis may be involved in pain generation (17, 19). Patients with the highest pain scores displayed significantly more neural invasion into endometriosis than those with lower pain scores (20). Therefore, less innervation may ameliorate the symptoms of disease. Tokushige et al. (21) reported that the nerve fiber density in peritoneal endometriotic lesions from women with endometriosis who were on hormone treatment with progestogens and combined oral contraceptives was statistically significantly lower than in peritoneal endometriotic lesions from untreated women with endometriosis. In the present study, our results showed that there was lower number of nerve fibers in the treatment group, which is consistent with the findings of previous studies.

The pathogenesis of endometriosis and pathophysiological basis for endometriosis–associated pain are still unclear. Endometriosis is believed to be a chronic in.ammatory state, with disturbances of both cell-mediated and humoral immunity (16). In women with endometriosis, the peritoneal .uid has high concentrations of cytokines, growth factors, and angiogenic factors (16, 22-24), derived from the lesions themselves; secretory products of macrophages and other immune cells; and follicular .uid after follicle rupture in ovulating women. Once endometriotic lesions are formed, they secrete several pro-in.ammatory molecules (23, 24).

These nerve fibers in endometriotic lesions probably play an important role in the pathogenesis of pain and hyperalgesia. The nerve endings of nerve fibers can potentially be stimulated by many inﬂammatory substances, including histamine, serotonin, bradykinin, prostaglandins, leukotrienes, interleukins (ILs), acetylcholine, VEGF, tumor necrosis factor-α (TNF-α), epidermal growth factors, transforming growth factor-β (TGF-β), platelet-derived growth factor and NGF. Many of the above substances can be secreted by macrophages as well as from endometriotic lesions (25, 26), and are found in high concentration in the peritoneal fluid of endometriosis patients. Moreover, macrophages and their products may play important roles in the growth and repair of nerve fibers. The growth of nerve fibers is regulated by many substances, including NGF, brain-derived neurotropic factor (BDNF) and VEGF, and the synthesis of these substances is also affected by macrophage activities.

HMSCs, first described by Fridenstein et al. (27) in 1974, have extensive proliferative potential and the capacity to differentiate into various cell types. The bone marrow has been considered as the major source of MSCs. Transplantation of bone marrow-MSCs (BM-MSCs), however, may not be acceptable because of the variations in cell numbers and the proliferative potential of these cells from different donors (28). Other sources of MSCs have been considered and currently the presence of MSCs has been confirmed in the placenta, amniotic ﬂuid, peripheral blood, lungs and teeth (29). Because there are large numbers of MSCs in neonates (30), human umbilical cords may be an ideal source for these cells. Supporting their potential as a source of cells, MSCs have been isolated from human umbilical cord (9, 27, 31). MSCs are poor antigen-presenting cells and do not express major histocompatibility complex class II or costimulatory molecules. HMSCs suppress T-lymphocyte proliferation induced by cellular or non-specific mitogenic stimuli and inhibit the response of naive and memory antigen-specific T cells to their cognate peptide (32). HMSCs altered the cytokine secretion profile of dendritic cells (DCs), naive and effector T cells [T helper 1 (T(h)1) and T(h)2], and natural killer (NK) cells to induce a more antiinﬂammatory or tolerant phenotype (33). MSCs have potent anti-inflammatory effects in multiple disease states (34). Some researchers have reported that MSCs administered by intravenous injections potently inhibit systemic levels of inflammatory cytokines and chemokines in the serum of treated animals (35). In addition, MSCs were able to modulate the immune system through the release of anti-inflammatory cytokines, prostaglandin E2 in many models (36). Aggarwal and Pittenger (33) reported that through the interactions of hMSCs with the various immune cells, hMSCs could inhibit or limit inﬂammatory responses and promote the mitigating and anti-inﬂammatory pathways. They demonstrated that when hMSCs are present in an inﬂammatory environment (such as that artificially created by activating DCs, macrophages, NK cells, or T cells using various stimuli), they may alter the outcome of the on-going immune response by altering the cytokine secretion profile of DC subsets (DC1 and DC2) and T-cell subsets (TH1, TH2, or TRegs), thereby resulting in a shift from a proinﬂammatory environment toward an anti-inﬂammatory or tolerant cell environment.

There was significantly lower number of nerve fibers stained with specific markers we used in the treatment group than in the control group. Endometriosis is a benign oestrogen-dependent inﬂammatory disease and hUC-MSCs could attune inflammatory effects of inflammatory factors such as cytokines, growth factors, and angiogenic factors. Other underlying mechanisms such as the differentiation of hUC-MSCs and/or the paracrine mediator secreted by hUC-MSCs may be also involved. A recent study also suggested that hUC -MSCs may serve as a promising treatment approach to ameliorate endometrial damage (37). Our study was the preliminary exploration of hUC –MSC treatment with endometriosis. The exact mechanism and outcome of hUC-MSCs remain to be elucidated in future studies.

## Conclusion

We demonstrated that hUC-MSCs could reduce nerve fibers density in the treatment group and may provide a new potential therapeutic modality to endometriosis.
